# Nuanced differences in adenylate cyclase toxin production, acylation, and secretion may contribute to the evolution of virulence in *Bordetella* species

**DOI:** 10.1128/mbio.01082-25

**Published:** 2025-05-19

**Authors:** Alexa R. Wolber, Liliana S. McKay, Katlyn B. Mote, Richard M. Johnson, Carol S. Inatsuka, Peggy A. Cotter

**Affiliations:** 1 Department of Microbiology and Immunology, School of Medicine, University of North Carolina-Chapel Hill 318275, Chapel Hill, North Carolina, USA; 2 Department of Molecular, Cellular and Developmental Biology, University of California 8786, Santa Barbara, California, USA; St Jude Children's Research Hospital, Memphis, Tennessee, USA

**Keywords:** *Bordetella*, adenylate cyclase toxin, acylation, bacterial respiratory infection, post-translational modification, *Bordetella pertussis*, *Bordetella bronchiseptica*

## Abstract

*Bordetella pertussis*, which causes the acute human disease whooping cough, evolved from *Bordetella bronchiseptica*, which causes long-term, chronic infections in a broad range of mammals. Both *B. pertussis* and *B. bronchiseptica* produce adenylate cyclase toxin (ACT), a bifunctional protein with adenylate cyclase-mediated cell intoxication and pore-forming activities. CyaC-mediated acylation of ACT is important for cell intoxication and required for pore-forming activity *in vitro*, but its role *in vivo* was unknown. Our comparative analysis showed that although ACT secreted by *B. pertussis* is fully acylated at residues K860 and K983, only a fraction of ACT secreted by *B. bronchiseptica* is modified. We showed that *B. bronchiseptica* produces more ACT than *B. pertussis* and is more efficient at ACT-dependent intoxication of macrophages *in vitro* than *B. pertussis*, but for both organisms, acylation of ACT greatly enhances intoxication. Acylation also enhances ACT secretion. Using a natural-host model, we determined that non-acylated ACT is functional during the initial stage of *B. bronchiseptica* infection, but not at later time points, and that acylation of K860, but not K983, is required for persistence in the lower respiratory tract. These data indicate a role for both acylated and non-acylated ACT during infection. Acylation of ACT was similarly not absolutely required for *B. pertussis* persistence in the murine lower respiratory tract. Overall, our data revealed nuanced differences in production, acylation, and secretion of ACT between *B. pertussis* and *B. bronchiseptica* that may correlate with their different virulence strategies.

**IMPORTANCE:**

*Bordetella pertussis* causes the acute disease whooping cough and survives only in the human respiratory tract, while *Bordetella bronchiseptica* causes long-term, chronic infections in a broad range of mammals and can also survive in extra-host environments. These bacteria produce a nearly identical set of virulence factors, including adenylate cyclase toxin (ACT), a protein that is modified by the addition of acyl chains. Acylation is required for ACT to cause hemolysis and for efficient intoxication of host cells *in vitro*. We found that ACT acylation is also important, but not absolutely required, during infection. We also discovered differences in ACT production, acylation, and secretion between *B. bronchiseptica* and *B. pertussis* that may contribute to the different virulence strategies of these species. This study highlights the advantage of conducting comparative analyses between closely related species to better understand the evolution of virulence.

## INTRODUCTION

Despite widespread vaccination, the acute disease whooping cough (or pertussis) remains a global health threat, especially for young children and infants who are susceptible to severe or fatal disease (1–3). The causative agent, *Bordetella pertussis*, is a gram-negative bacterium that survives only within the human respiratory tract and briefly during transmission from host to host. Closely related *Bordetella bronchiseptica* infects nearly all mammals and causes a chronic and typically mild or asymptomatic disease (4). Unlike *B. pertussis*, *B. bronchiseptica* is also able to survive in extra-host environments. Phylogenetic analyses estimate that *B. pertussis* evolved from a *B. bronchiseptica*-like ancestor around 3 million years ago (5–7).

Although *B. pertussis* and *B. bronchiseptica* differ in virulence, both produce a similar set of virulence factors, including adenylate cyclase toxin (ACT). ACT is a bifunctional toxin with enzymatic adenylate cyclase and pore-forming activities. The N-terminal adenylate cyclase (AC) domain (~380 residues) binds calmodulin upon delivery to host cells and catalyzes an unregulated conversion of cellular ATP to cyclic AMP (cAMP), resulting in supraphysiological cAMP levels in a process referred to as cell intoxication. This rapid intoxication almost immediately suppresses oxidative burst, chemotaxis, and phagocytosis in neutrophils and macrophages (8–11).

The C terminus of ACT consists of a ~1,300 residue repeats-in-toxin (RTX) domain and, like other RTX proteins, forms pores in membranes and has hemolytic activity (12–14). With high affinity, a region of the RTX domain between residues 1166 and 1281 binds the CD11b subunit of CD11b/CD18, aka complement receptor 3 (CR3), expressed on myeloid cells such as macrophages, neutrophils, and dendritic cells (15, 16). Binding of CR3 enhances delivery of the AC domain to the host cell cytosol and subsequent cell intoxication (17).

RTX domain-mediated cytolysis depends on post-translational modifications (PTMs) for activation. ACT first synthesized as pro-CyaA (encoded by the *cyaA* gene) is modified by an acyltransferase, CyaC, at two internal lysine residues, K860 and K983 (18–20), before being secreted through a dedicated type 1 secretion system (T1SS; encoded by the *cyaBDE* genes) (12). ACT purified from culture supernatants of *B. pertussis* is nearly fully acylated, and the majority of PTMs detected at K860 and K983 were palmitoyl (C16:0) chains with a small proportion of myristoyl (C14:0) chains detected at K983 (19, 21).

Studies performed *in vitro* with purified toxin have shown that acylation of ACT is important, but not absolutely required, for intoxication of J774 macrophage-like cells (11, 16, 22). Acylation of ACT appears to be critical for cytolysis by pore formation in both CR3-negative cells (i.e., hemolysis of erythrocytes) and CR3-positive cells in a toxin concentration-dependent manner *in vitro (22–25*). Both enzymatic and pore-forming capacities of ACT further synergize to produce overall cytotoxic effects on CR3-positive cells *in vitro* (22, 26). However, these activities mediated by *Bordetella*-delivered ACT, as opposed to purified toxin, are less characterized.

In a natural-host model of infection, ACT and AC activity specifically have been shown to be critical for *B. bronchiseptica* persistence in the lower respiratory tract (LRT) of mice during the initial stage of infection (27, 28). Although natural-host models of infection for *B. pertussis* are not available, studies with mice have been done that suggest ACT is also important for *B. pertussis* persistence in the LRT (29–35). Based on *in vitro* studies, acylation of ACT has long been assumed to be essential for ACT activity *in vivo*. However, for both *B. bronchiseptica* and *B. pertussis*, the role of ACT acylation in persistence during infection has yet to be determined. Therefore, we conducted a comparative study to investigate the role of ACT acylation in *B. bronchiseptica* and *B. pertussis in vivo*.

## RESULTS

### Acylation is required for *B. bronchiseptica* and *B. pertussis* hemolysis

**Fig 1:**
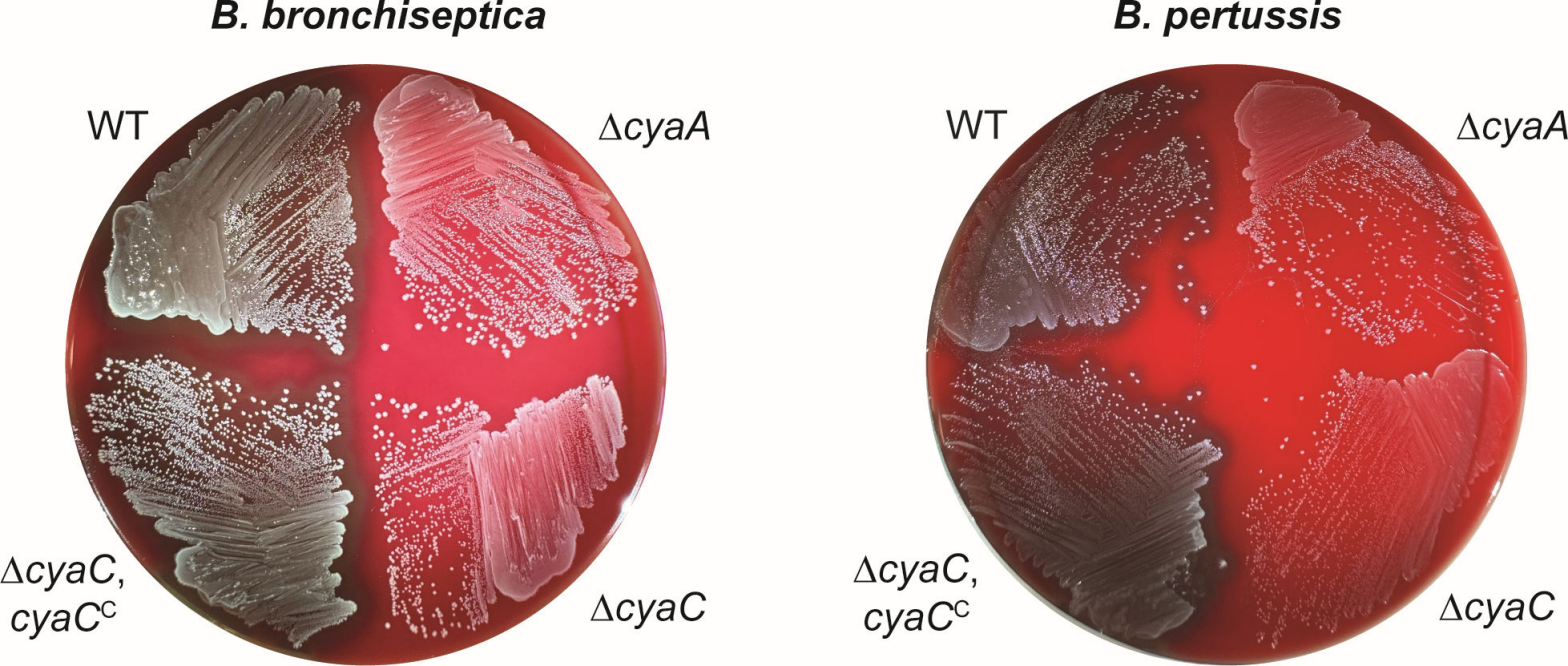
Acylation is required for *B. bronchiseptica* and *B. pertussis* ACT-dependent hemolysis on blood agar. Growth after 72 hours (*B. bronchiseptica* strains) or 5 days (*B. pertussis* strains) on Bordet-Gengou agar containing 12.5% defibrinated sheep’s blood. WT, wild type.

To investigate the role of ACT acylation in *Bordetella* virulence, we constructed strains with an in-frame deletion in the acyltransferase-encoding gene, *cyaC*, in *B. bronchiseptica* and *B. pertussis*. We also constructed *cyaC* complementation strains in which the *cyaC* gene and the entire upstream intergenic region containing its native promoter were inserted into the *att*Tn*7* site in the ∆*cyaC* strains (hereafter named ∆*cyaC*,*cyaC*^C^). For both *B. bronchiseptica* and *B. pertussis*, the ∆*cyaC* mutant was non-hemolytic on blood agar plates, similar to ACT-deficient (∆*cyaA*) strains, and hemolysis was restored by complementation in the ∆*cyaC*,*cyaC*^C^ strains (Fig. 1).

### More ACT is produced, secreted, and surface-associated in *B. bronchiseptica* than in *B. pertussis*

**Fig 2:**
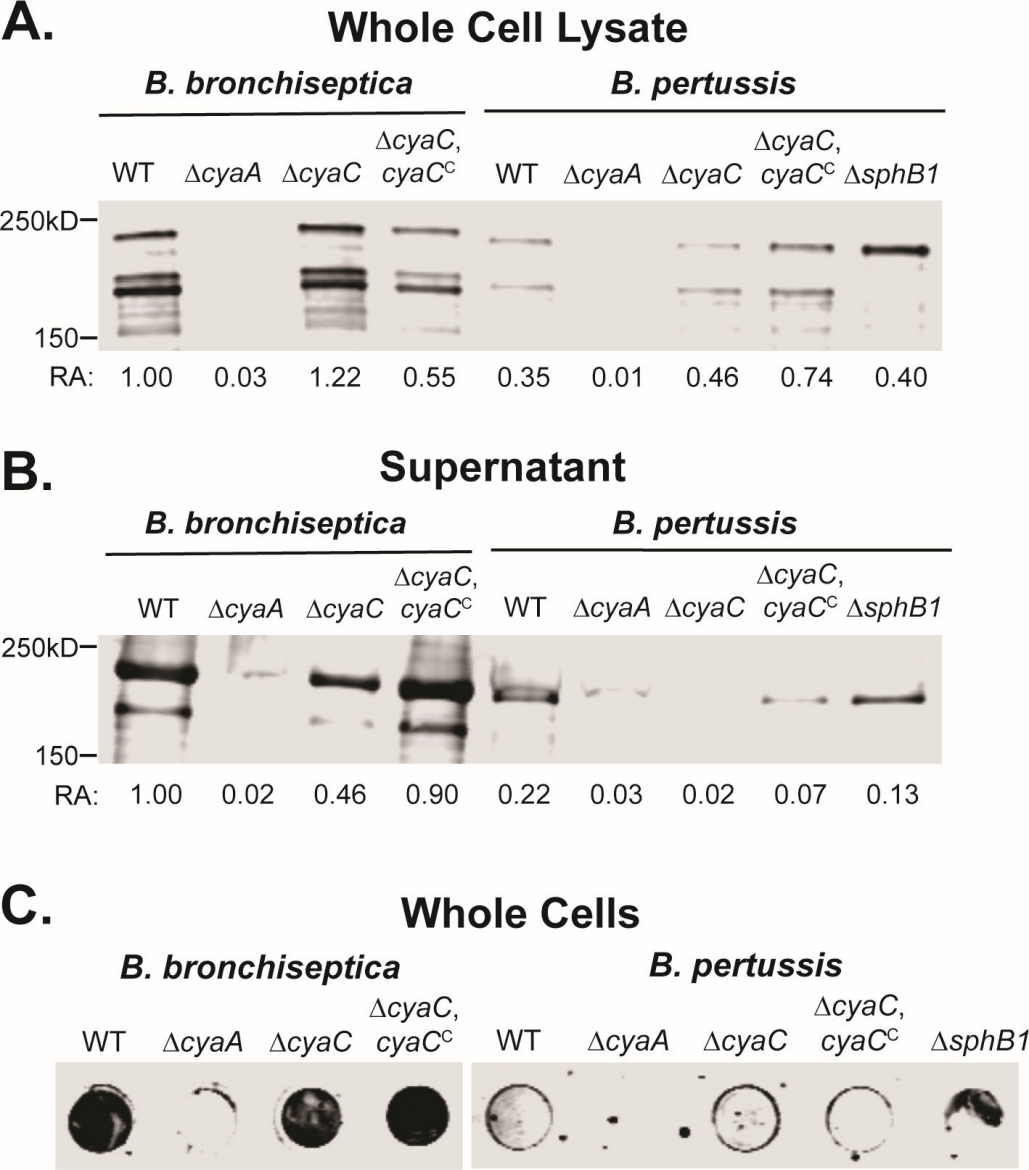
ACT production, secretion, and surface association are greater in *B. bronchiseptica* than in *B. pertussis*, and acylation enhances secretion. Western blot analyses of whole cell lysates (**A**) or supernatants (**B**) collected from *B. bronchiseptica* and *B. pertussis* strains grown in SS medium supplemented to 2 mM CaCl_2_. Samples were taken after 16 hours of growth and normalized to culture OD_600_. Blots were probed with monoclonal α-ACT antibody. Relative abundance (RA) values under each lane correspond to quantified ACT by normalizing ACT signal to the signal of a protein of relative equal abundance identified by Revert 700 Total Protein Stain (LI-COR) present in whole cell lysates across all strains. (**C**) Dot blot analyses of intact bacteria that were cultured, normalized, and probed as described for panels **A** and **B**. WT, wild type.

To determine ACT production and secretion in *B. bronchiseptica* and *B. pertussis*, we compared wild-type and mutant strains by western blot analyses after growth in modified Stainer-Scholte (SS) medium containing 2 mM Ca^2+^, which has been demonstrated to alter ACT localization by accelerating ACT translocation through its T1SS (36, 37). To quantify ACT protein abundance in whole cell lysates (WCL) and supernatants from overnight cultures grown to a similar OD_600_, we normalized ACT signal to the signal of a protein in WCL that was of equal abundance across all strains. With this normalization, more ACT was present in WCL and culture supernatants of wild-type *B. bronchiseptica* compared to wild-type *B. pertussis* (Fig. 2A and B). These data indicate *B. bronchiseptica* produces and secretes more ACT than *B. pertussis* under these conditions.

To determine the surface association of ACT in *B. bronchiseptica* and *B. pertussis*, we spotted intact whole cells from overnight cultures grown in modified SS medium containing 2 mM Ca^2+^ on membranes and used immunoblot analyses to detect ACT. ACT was detectable on the cell surface of wild-type *B. bronchiseptica*, but was barely detectable on the cell surface of wild-type *B. pertussis* (Fig. 2C), despite ACT being present in WCL and supernatants (Fig. 2A and B). These data indicate that in *B. bronchiseptica*, a substantial amount of ACT is surface-associated, but in *B. pertussis*, hardly any secreted ACT remains surface-associated. These data are consistent with the results of both Bumba et al. and Nash et al.; when grown in SS medium containing 2 mM Ca^2+^, ACT remains cell surface-associated in *B. bronchiseptica* but minimally in *B. pertussis* (36, 37).

### Acylation facilitates secretion of ACT

To determine if acylation is required for production, secretion, or surface association of ACT in *B. bronchiseptica* and *B. pertussis*, we compared wild-type and mutant strains by western blot and dot blot analyses. In both *B. bronchiseptica* and *B. pertussis*, ACT was detected at similar levels in WCL of wild-type, ∆*cyaC*, and ∆*cyaC*,*cyaC*^C^ strains (Fig. 2A). ACT was less abundant in supernatants of the ∆*cyaC* mutants compared to wild-type and ∆*cyaC*,*cyaC*^C^ strains (Fig. 2B), suggesting that acylation facilitates secretion of ACT.

ACT was detectable on the cell surface of wild-type, ∆*cyaC*, and ∆*cyaC*,*cyaC*^C^ *B. bronchiseptica*, but hardly any was detectable on the cell surface of *B. pertussis* wild-type, ∆*cyaC*, and ∆*cyaC*,*cyaC*^C^ strains (Fig. 2C), despite ACT being present in WCL and supernatants (Fig. 2A and B), indicating that acylation does not affect surface association of ACT.

### ACT cleavage is SphB1-dependent in *B. pertussis*

In *B. bronchiseptica*, ACT is cleaved in a manner dependent on the surface autotransporter serine protease SphB1 (36). To determine if ACT cleavage is SphB1-dependent in *B. pertussis*, we compared ACT production and secretion by western blot analysis in wild-type and ∆*sphB1 B. pertussis* strains. Only full-length ACT was detected in whole cell lysates and supernatants in the ∆*sphB1* mutant (Fig. 2A and B, right). Similar to wild-type, ∆*cyaC*, and ∆*cyaC*,*cyaC*^C^ *B. pertussis* strains, hardly any ACT was detectable on the cell surface of ∆*sphB1* bacteria (Fig. 2C, right). These data indicate ACT cleavage is SphB1-dependent in *B. pertussis* and that SphB1-dependent cleavage does not affect the localization of ACT.

### Acylation of ACT enhances, but is not essential for, host cell intoxication by *B. bronchiseptica* and *B. pertussis*

**Fig 3:**
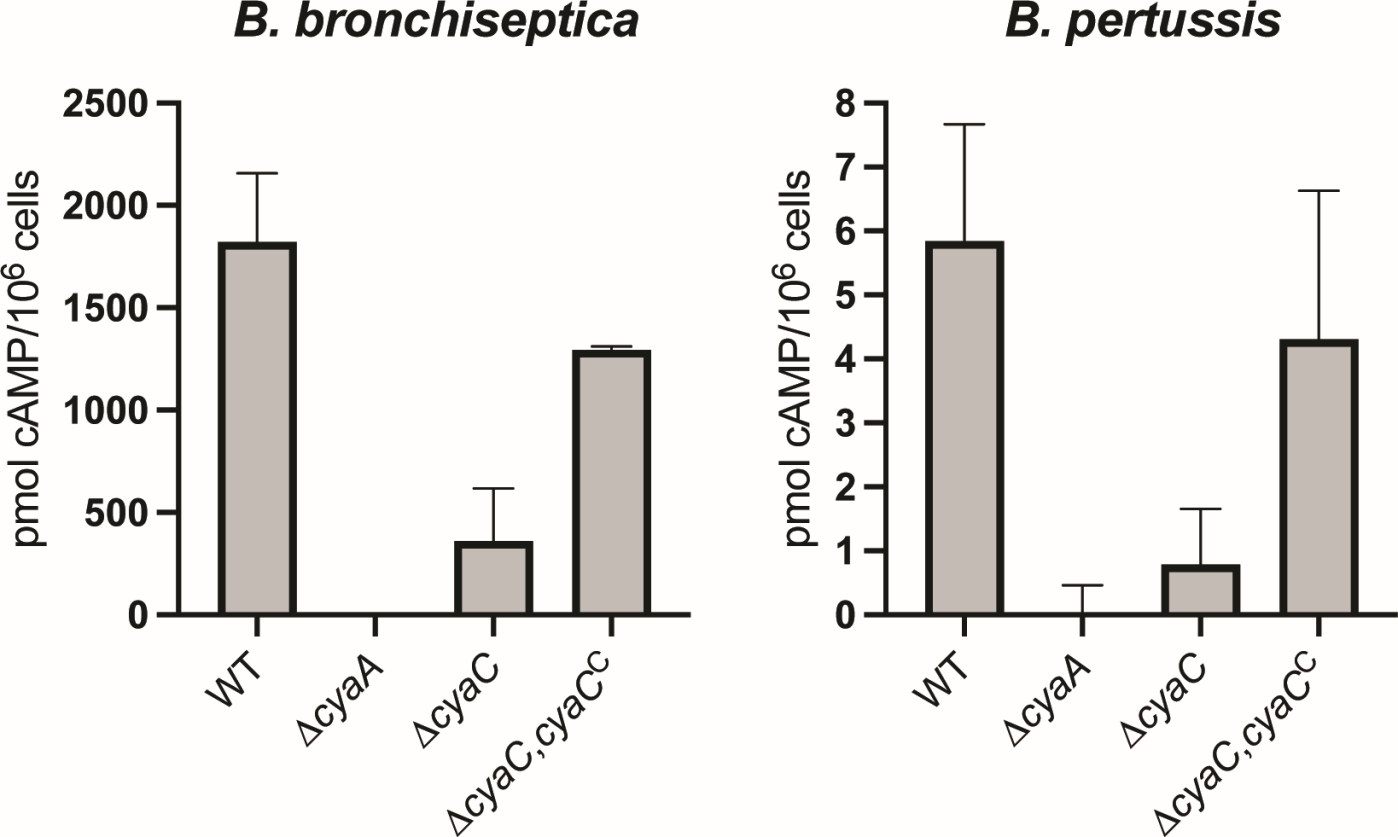
ACT acylation enhances *B. bronchiseptica* and *B. pertussis* intoxication of J774A.1 murine macrophage-like cells. J774A.1 cells were infected with *B. bronchiseptica* and *B. pertussis* wild-type (WT) or mutant strains at an MOI of 100 for 30 minutes. Intracellular cAMP concentrations were determined by ELISA, and the concentration of cAMP for the uninfected control was subtracted from the experimental samples. The data shown are compiled from four biologically independent experiments for *B. bronchiseptica* WT and ∆*cyaC* strains and two biologically independent experiments for all other *B. bronchiseptica* and *B. pertussis* strains.

To determine if acylation is required for host cell intoxication, we infected J774A.1 murine macrophage-like cells with wild-type and mutant *B. bronchiseptica* and *B. pertussis* strains at a multiplicity of infection (MOI) of 100 and measured 3′−5′-cAMP levels by enzyme-linked immunosorbent assay (ELISA). As previously shown (36), wild-type *B. bronchiseptica* induced a dramatic increase in cAMP production, whereas the ∆*cyaA* mutant did not (Fig. 3, left). The ∆*cyaC* mutant induced a slight increase in cAMP production, but much less than that induced by the wild-type strain (Fig. 3, left). Complementation (∆*cyaC*,*cyaC*^C^) restored cAMP production to wild-type levels (Fig. 3, left).

Wild-type *B. pertussis* also induced cAMP production in J774A.1 cells (Fig. 3, right), but the increase was not nearly as dramatic as that induced by *B. bronchiseptica*, which is consistent with more ACT being produced and secreted by *B. bronchiseptica* than *B. pertussis* (Fig. 2B). No increase in cAMP was induced by the ∆*cyaA B. pertussis* mutant. The ∆*cyaC* mutant induced a slight increase in cAMP, but much less than that induced by the wild-type strain (Fig. 3, right). Complementation (∆*cyaC*,*cyaC*^C^) restored cAMP production to wild-type levels (Fig. 3, right). Together, these data indicate that for both *B. bronchiseptica* and *B. pertussis*, ACT acylation greatly enhances, but is not fully required for, intoxication of J774A.1 macrophage-like cells.

### ACT secreted by *B. bronchiseptica* is only partially acylated

**Fig 4:**
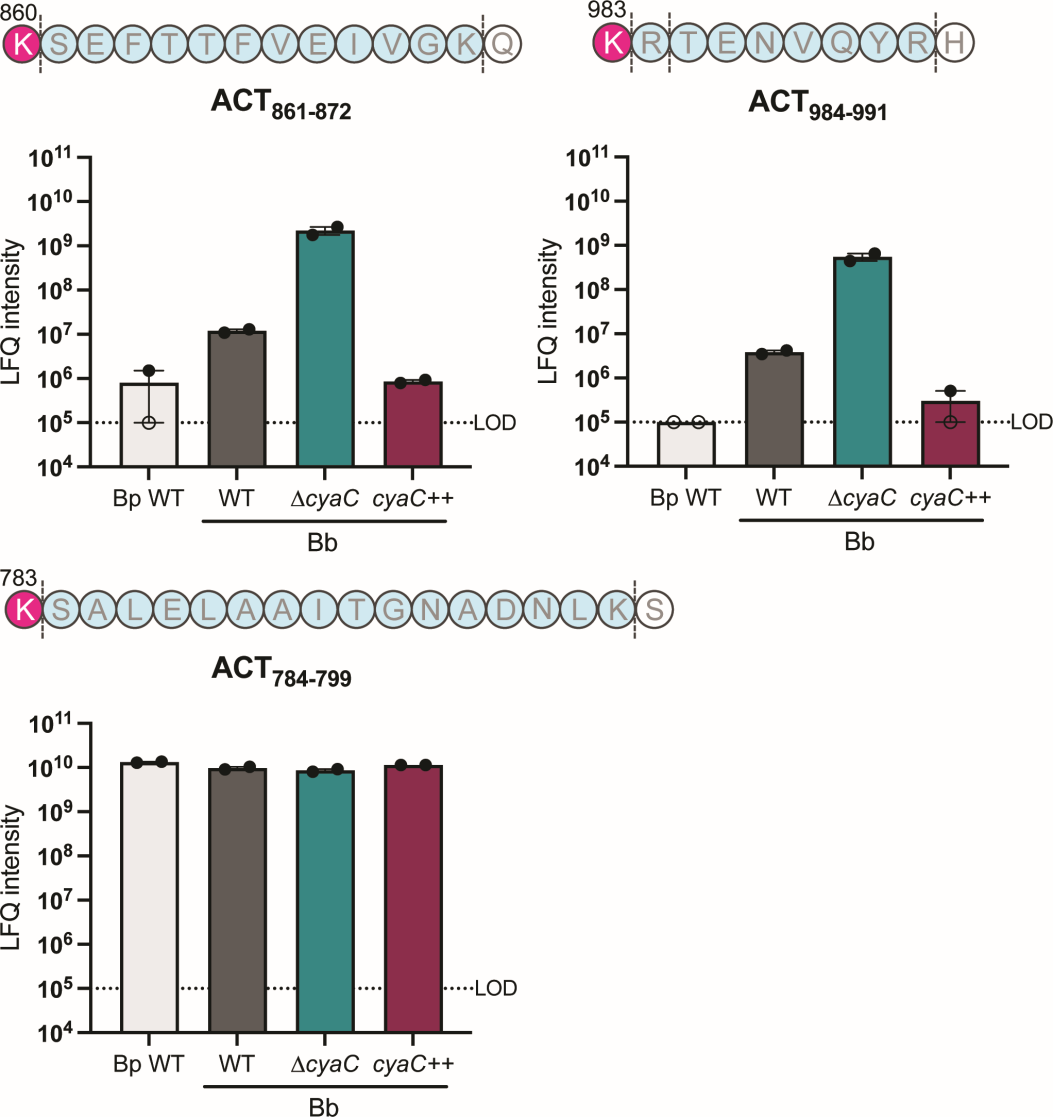
Only a fraction of ACT secreted by *B. bronchiseptica* is modified at K860 and K983. Linear representations of the peptide sequences following residues K860, K983, and K783 of ACT. Dashed lines indicate trypsin digestion sites. Sequences in blue between dashed lines represent the peptides that were quantified by LC-MS/MS. Open circles represent no peptide detected in the sample and plotted at the limit of detection. Relative abundances (LFQ intensities) were corrected using global normalization to compare across samples. WT, wild type.

Previous studies have shown that nearly 100% of the ACT secreted by *B. pertussis* is acylated at residues K860 and K983 (19, 21). While K860 appears to be only palmitoylated, both palmitoylation and myristoylation of K983 were detected (21). To determine the level of acylation of ACT secreted by *B. bronchiseptica*, we took a mass spectrometry (liquid chromatography-mass spectrometry/mass spectromerty [LC-MS/MS]) approach. Because our instrumentation could not directly detect the predicted PTMs, we instead measured peptide abundance as evidence of modification. Since trypsin cleaves C-terminal to lysine and arginine residues and modification of lysine or arginine prevents trypsin cleavage, the abundance of peptides generated by trypsin digestion directly reflects residue modification. For example, the abundance of ACT_861-872_ reflects the level of modification of K860 (Fig. 4), in which a high abundance (label-free quantification [LFQ] intensity) indicates effective trypsin cleavage at K860 when the site is unmodified. To avoid cleavage of ACT by SphB1 and interference from FhaB/FHA, we collected ACT from supernatants of ∆*sphB1*∆*fhaB B. pertussis* and *B. bronchiseptica* strains. For *B. bronchiseptica*, we also included ∆*sphB1*∆*fhaB* derivatives in which *cyaC* was deleted (∆*cyaC*) or *cyaC* driven by the strong constitutive S12 promoter was present at the *att*Tn*7* site (*cyaC*++). For *B. pertussis*, the abundances of ACT_861-872_ and ACT_984-991_ were at or close to the lower limit of detection (Fig. 4), consistent with nearly all the ACT secreted by *B. pertussis* being modified at both K860 and K983. For ACT from wild-type *B. bronchiseptica*, the abundances of ACT_861-872_ and ACT_984-991_ were approximately 10^7^ LFQ intensity units (Fig. 4). For ACT from ∆*cyaC B. bronchiseptica*, the abundances of ACT_861-872_ and ACT_984-991_ were approximately 10^9^ LFQ intensity units, and for ACT from the *cyaC*++ strain, the abundances were at 10^6^ LFQ intensity units or below the limit of detection (Fig. 4). For comparison, the abundance of ACT_784-799_ was at 10^10^ LFQ intensity units in all strains, indicating effective trypsin cleavage at K783, which is not predicted to be modified (Fig. 4). The fact that ACT_861-872_ and ACT_984-991_ abundances were approximately 2 logs lower in wild-type *B. bronchiseptica* than the ∆*cyaC* strain, in which ACT cannot be acylated, indicates that ACT from wild-type *B. bronchiseptica* is at least partially modified at K860 and K983. However, the fact that ACT_861-872_ and ACT_984-991_ abundances were 1–2 logs greater in wild-type *B. bronchiseptica* compared to the *cyaC*++ and wild-type *B. pertussis* strains indicates that ACT is not fully modified at K860 and K983 in wild-type *B. bronchiseptica*. Overall, these data indicate that only a fraction of ACT secreted by wild-type *B. bronchiseptica* is modified at K860 and K983.

### Both acylated and non-acylated ACT contribute to *B. bronchiseptica* persistence in the lower respiratory tract

**Fig 5:**
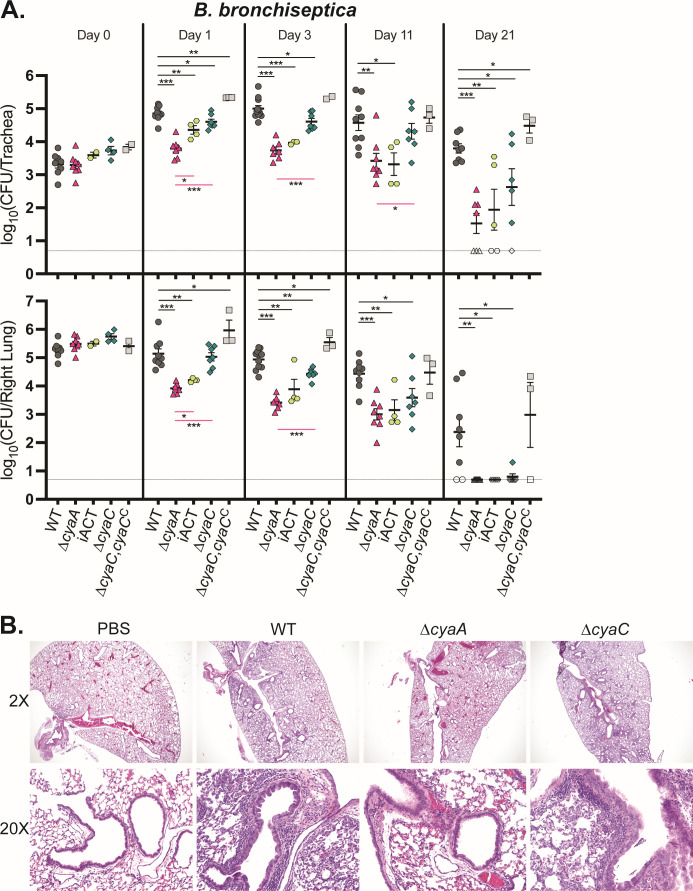
Acylated and non-acylated ACT are functional during *B. bronchiseptica* infection. (**A**) Bacterial burden over time in the trachea (top) and right lung (bottom) of mice inoculated with wild-type (WT) *B. bronchiseptica* or mutant strains. Data were compiled from four independent experiments, each including WT. Dashed line represents the limit of detection. Only statistically significant differences in bacterial burden compared to WT (above; black lines) or ∆*cyaA* (below; pink lines) are reported as determined using unpaired Student’s *t*-test; *P < 0.05, **P < 0.01, ***P < 0.001. (**B**) Representative images of hematoxylin-and-eosin-stained 5 µm left lung sections from day 3 mice inoculated with WT or mutant strains in panel **A**.

Based on *in vitro* studies, we hypothesized that acylation of ACT would be critical for *Bordetella* spp. persistence during infection. To determine the role of acylation during *B. bronchiseptica* infection, we used a high-dose, large-volume murine model of infection. After intranasal inoculation, we determined bacterial burden in the trachea and right lung lobes at various time points. As previously shown (28), the ∆*cyaA* and catalytically inactive (iACT) mutants were defective for persistence relative to wild-type bacteria in both the trachea and lung at day 1 post-inoculation and continued to be defective at all time points (Fig. 5A). Surprisingly, the ∆*cyaC* mutant was recovered at levels similar to those of the wild-type strain from the lungs and trachea at day 1 post-inoculation (Fig. 5A). At day 3, the ∆*cyaC* mutant was slightly defective for persistence in both the trachea and lung relative to wild-type bacteria, but not as defective as the ∆*cyaA* mutant (Fig. 5A). By day 11, the ∆*cyaC* mutant was as defective as the ∆*cyaA* mutant for persistence in the lung and was eventually cleared from the lung by day 21 (Fig. 5A). Complementation (∆*cyaC*,*cyaC*^C^) restored bacterial persistence in the trachea and lung to wild-type levels at all time points (Fig. 5A). These results indicate non-acylated ACT is functional during the initial stage of infection, and that acylated ACT is required for bacterial persistence at later time points.

To evaluate the role of ACT acylation on lung inflammation during *B. bronchiseptica* infection, we compared hematoxylin-and-eosin (H&E)-stained left lung sections from the mice we recovered colony forming units (CFU) from at day 3 post-inoculation. Lungs from phosphate-buffered saline (PBS)-inoculated mice showed no observable signs of inflammatory cell infiltrate, as expected (Fig. 5B). Mild inflammation was observable primarily around the large airways of the lung in mice inoculated with wild-type *B. bronchiseptica* (Fig. 5B). By contrast, the lungs of ∆*cyaA*-inoculated mice had significantly less cellular infiltrate (Fig. 5B). The lungs of mice inoculated with the ∆*cyaC* mutant showed similar levels of inflammation localized around the large airways as lungs of mice inoculated with wild-type bacteria (Fig. 5B). These data further suggest non-acylated ACT is functional during the initial stage of *B. bronchiseptica* infection, and the level of cellular infiltrate is consistent with the ∆*cyaC* bacterial burden being closer to the wild-type bacterial burden in the lung.

### Acylation of ACT residue K860, but not K983, is important for *B. bronchiseptica* persistence in the lower respiratory tract

**Fig 6:**
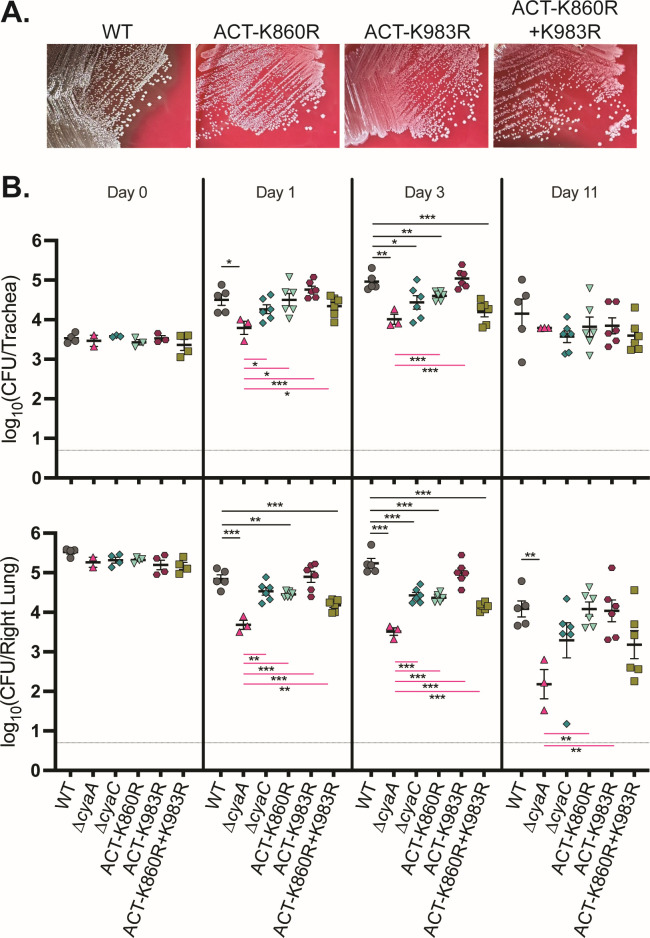
Acylation of ACT residue K860 is important for *B. bronchiseptica* persistence in the lower respiratory tract. (**A**) Growth after 72 hours on Bordet-Gengou agar containing 6% defibrinated sheep’s blood. (**B**) Bacterial burden over time in the trachea (top) and right lung (bottom) of mice inoculated with *B. bronchiseptica* wild-type (WT) or mutant strains. Data were compiled from two independent experiments. Dashed line represents the limit of detection. Only statistically significant differences in bacterial burden compared to WT (above; black lines) or ∆*cyaA* (below; pink lines) are reported as determined using unpaired Student’s *t*-test; *P < 0.05, **P < 0.01, ***P < 0.001.

While there are no other known CyaC substrates, we sought to determine if the defect of the *B. bronchiseptica* ∆*cyaC* mutant in persistence during infection was due to the lack of acylation of ACT residues K860 and/or K983. We constructed *B. bronchiseptica* strains producing ACT with amino acid substitutions at each residue individually (ACT-K860R and ACT-K983R) or at both sites (ACT-K860R+K983R). ACT-K860R, ACT-K983R, and ACT-K860R+K983R were all non-hemolytic on blood agar plates (Fig. 6A), demonstrating that acylation of both K860 and K983 is required for pore formation in erythrocytes. We inoculated mice with these mutants and determined bacterial burden in the trachea and right lung at various time points. At days 1 and 3 post-inoculation, the ACT-K860R and ACT-K860R+K983R mutants were indistinguishable from the ∆*cyaC* mutant for persistence in the lung, while the ACT-K983R mutant was indistinguishable from the wild-type strain (Fig. 6B). These data indicate K860 is the primary site of acylation required for bacterial persistence and confirm that ACT is the only CyaC substrate that plays a role in bacterial persistence during infection. Statistically significant differences were less apparent in this experiment on day 11, likely due to an increase in variation within the data set and a decrease in the number of mice (Fig. 6B). However, these results do not alter the conclusion that acylation of ACT (at K860) is important, but not absolutely required, for ACT-mediated persistence in the lower respiratory tract.

### Acylation is only partially required for ACT-mediated persistence in the lower respiratory tract in *B. pertussis*

**Fig 7:**
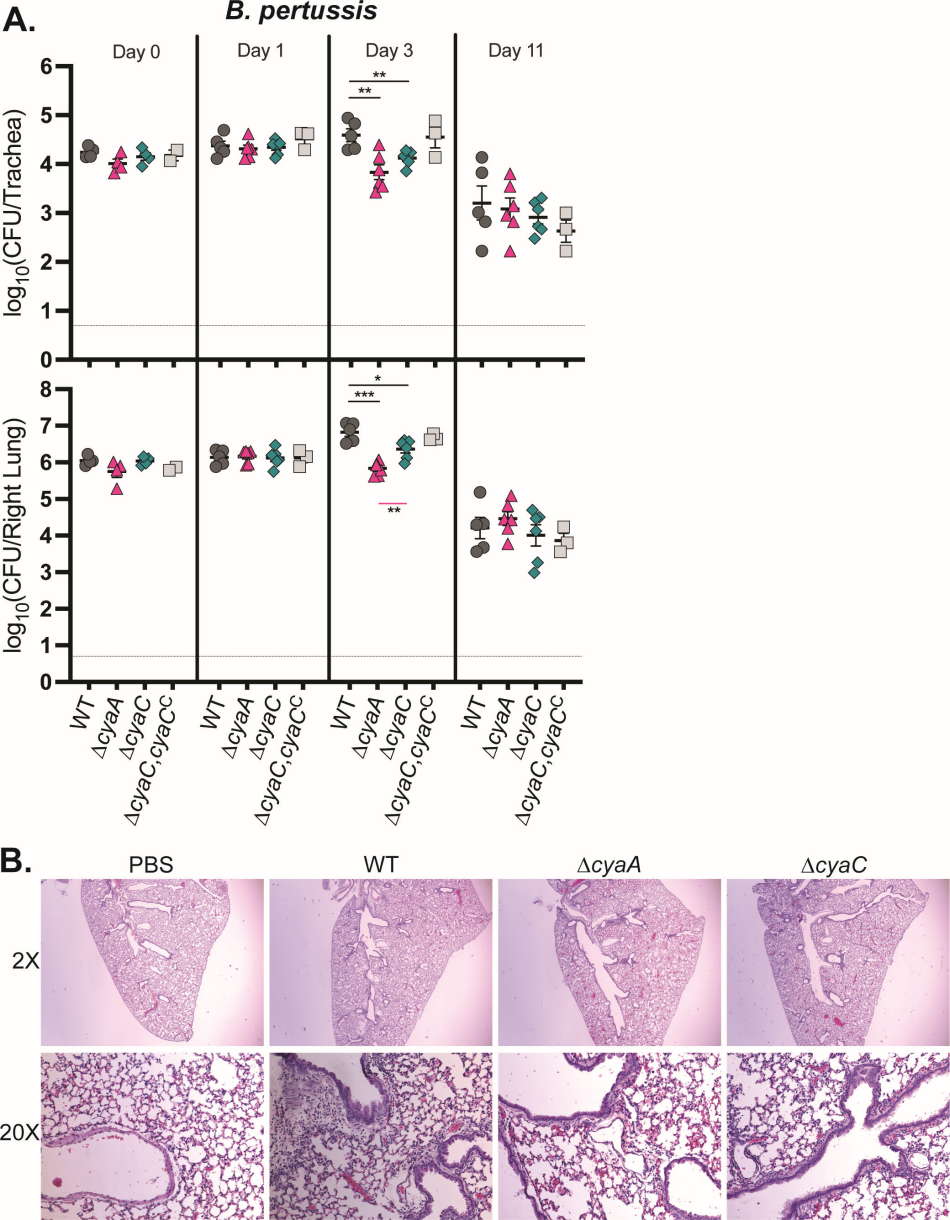
Acylation is only partially required for *B. pertussis* persistence in mice. (**A**) Bacterial burden over time in the trachea (top) and right lung (bottom) of mice inoculated with *B. pertussis* wild-type (WT) or mutant strains. Data were compiled from two independent experiments. Dashed line represents the limit of detection. Only statistically significant differences in bacterial burden compared to WT (above; black lines) or ∆*cyaA* (below; pink lines) are reported as determined using unpaired Student’s *t*-test; *P < 0.05, **P < 0.01, ***P < 0.001. (**B**) Representative images of H&E-stained 5 µm left lung sections from day 3 mice inoculated with PBS vehicle control, WT, or mutant strains in panel **A**.

To investigate the role of acylation during *B. pertussis* infection, we used our high-dose, large-volume murine model of infection. In contrast to *B. bronchiseptica*, the *B. pertussis* ∆*cyaA* mutant was only slightly defective for persistence in the trachea and lungs at day 3 post-inoculation (Fig. 7A), suggesting that ACT plays a more minor role in *B. pertussis* infection compared to *B. bronchiseptica*, at least in this animal model. Similar to the case with *B. bronchiseptica*, however, the ∆*cyaC* mutant was recovered at numbers intermediate between wild-type and ∆*cyaA* bacteria at day 3, indicating that acylation is important, but not absolutely required for ACT-mediated persistence in the LRT in *B. pertussis*, similar to *B. bronchiseptica*. Complementation in the ∆*cyaC*,*cyaC*^C^ strain restored bacterial persistence in the trachea and lung back to wild-type levels (Fig. 7A). To evaluate the role of ACT acylation in lung inflammation, we examined H&E-stained left lung sections recovered from mice at day 3 post-inoculation. PBS-inoculated mice showed no observable signs of inflammatory cell infiltrate at this time point (Fig. 7B), and no observable increase in cellular infiltrate was present in the lungs of mice inoculated with *B. pertussis* wild-type, ∆*cyaA*, or ∆*cyaC* compared to PBS-inoculated mice (Fig. 7B). These data suggest that despite the high inoculum, *B. pertussis* does not induce a strong inflammatory response in mice at day 3 post-inoculation, the only time point at which we observed a role for ACT in bacterial persistence.

## DISCUSSION

While the purpose of this study was to understand the role of ACT acylation in *Bordetella* virulence, our results also revealed nuanced differences between *B. bronchiseptica* and *B. pertussis* that inform a better understanding of the evolution of virulence in these species.

One of our first observations relates to contradictory reports regarding the association of ACT on the *Bordetella* cell surface. By measuring adenylate cyclase activity of culture supernatants versus intact cells of *B. pertussis* grown in standard Stainer-Scholte medium containing 0.18 mM Ca^2+^, Hewlett et al. first reported that the majority of secreted ACT remains associated with the bacterial cell surface (38). Using overlay and immunoprecipitation, the Hewlett lab further demonstrated that ACT remains surface-associated via interactions with filamentous hemagglutinin (FhaB/FHA) (39). By measuring adenylate cyclase activity of urea extracts from *B. pertussis* cultures grown in SS containing 0.18 mM Ca^2+^ versus SS containing 2 mM Ca^2+^, Bumba et al. reported that nearly all ACT secreted by *B. pertussis* grown in SS containing 2 mM Ca^2+^ is released into culture supernatants, suggesting that surface retention of ACT is an artifact of growing the bacteria in 0.18 mM Ca^2+^ (37). Using western blot and dot blot analyses, our lab recently reported that while more ACT is detected in culture supernatants of *B. bronchiseptica* grown in SS containing 2 mM Ca^2+^ compared to SS containing 0.18 mM Ca^2+^, enough ACT is still present on the bacterial surface of *B. bronchiseptica* grown in SS containing 2 mM Ca^2+^ to be detected by dot blot analysis (36). Here, we showed that ACT was barely detectable on the *B. pertussis* surface by dot blot analysis after growth in SS containing 2 mM Ca^2+^ (Fig. 2). Hence, the apparently contradictory results between the Hewlett, Sebo, and Cotter labs are not merely an artifact of different experimental approaches or of the labs conducting the experiments, but instead reflect biological differences between *B. pertussis* and *B. bronchiseptica* strains, at least those used in our analyses. How these results extend to other *B. pertussis* and *B. bronchiseptica* strains is currently unknown.

In this study, we also discovered that cleavage of ACT in *B. pertussis* is dependent on SphB1, as we recently showed for *B. bronchiseptica (36*). However, SphB1-dependent cleavage differs somewhat between *B. bronchiseptica* and *B. pertussis*. In *B. bronchiseptica*, SphB1-dependent cleavage of ACT occurs predominantly between residues L325 and T326 to generate polypeptides ~200 kD and ~175 kD, which are detectable in whole cell lysates and supernatants (36). In *B. pertussis*, the same size polypeptides are present in whole cell lysates but do not appear as predominant in culture supernatants as they do in *B. bronchiseptica* culture supernatants (Fig. 2). This difference could be due to ACT being more efficiently released from the cell surface in *B. pertussis* than in *B. bronchiseptica*, which would decrease the time that ACT is available to the surface-localized SphB1 protease.

Our analyses also indicated that *B. bronchiseptica* produces and secretes approximately fivefold more ACT than *B. pertussis* (Fig. 2B), and that the amount of cAMP produced in macrophages incubated with *B. bronchiseptica* was approximately 300-fold greater than in macrophages incubated with *B. pertussis* (Fig. 3). These apparently discordant results may simply reflect the fact that the cAMP measured in the intoxication assay is the product of an enzymatic reaction and not the amount of the enzyme itself, while the western blots are a direct measurement of the amount of protein present in the samples. However, it is also possible that *B. bronchiseptica* delivers ACT to macrophages more efficiently than *B. pertussis* by some unknown mechanism. In both *B. bronchiseptica* and *B. pertussis*, the ∆*cyaC* strain was defective for intoxication. This defect is not merely due to less ACT being secreted, however, because studies done with equal concentrations of purified acylated versus non-acylated ACT show a similar phenotype (11). Hence, acylation appears to facilitate, but is not absolutely required for, toxin delivery to host cells.

The mechanistic basis for ACT production differing between *B. bronchiseptica* and *B. pertussis* is unknown. In *B. bronchiseptica* and *B. pertussis*, the global two-component regulatory system, BvgAS, activates expression of all known protein virulence factor-encoding genes, including *cyaA*. Our lab previously showed that induction of *cyaA* expression occurs substantially earlier post-shift from Bvg^–^ mode to Bvg^+^ mode in *B. bronchiseptica* than in *B. pertussis*, and this difference in *cyaA* activation is not due to differences in the *cyaA* promoters (which are nearly identical) (40). The same study showed that a *B. bronchiseptica* strain with the *bvgAS* genes from *B. pertussis* also induced *cyaA* expression “early” post-shift from Bvg^–^ mode to Bvg^+^ mode conditions (40), despite the BvgAS systems displaying differences in signal sensitivity and responsiveness (41). Thus, it seems that BvgAS control of *cyaA* expression in *B. bronchiseptica* differs from BvgAS control of *cyaA* expression in *B. pertussis* in a manner that is independent of the *cyaA* promoters or the BvgAS systems themselves. Possible influencing factors are differences in the BvgAS regulons between the organisms or differences in physiology and metabolism. However, we also note that we measured protein levels in our study and not gene expression, and hence differences in ACT levels are not necessarily (solely) due to differences in *cyaA* expression.

In this study, we showed that only a fraction of ACT secreted by *B. bronchiseptica* is modified at K860 and/or K983 (Fig. 5), unlike the case in *B. pertussis*, where most, if not all, secreted ACT is acylated at both K860 and K983 (19, 21). At the amino acid level, CyaC is identical between *B. bronchiseptica* and *B. pertussis*, and the *cyaA* genes are highly similar and functionally interchangeable (28), suggesting the difference in ACT acylation between *B. bronchiseptica* and *B. pertussis* is not due to differences in CyaC or ACT at the sequence level. We hypothesize that the difference in ACT acylation could be due to differences in intracellular pro-CyaA concentrations. The increased amount of pro-CyaA in *B. bronchiseptica* could simply be more than CyaC can handle, resulting in the secretion of mostly partially modified or unmodified ACT. Consistent with this hypothesis, overexpression of *cyaC* in *B. bronchiseptica* caused most of the secreted ACT to be fully modified at K860 and K983 (Fig. 5).

Given the *in vitro* evidence suggesting that acylation is critical for ACT functionality (19, 23, 24), we were surprised that the ∆*cyaC* mutant did not phenocopy the ∆*cyaA* or iACT mutants during the initial stage of *B. bronchiseptica* infection, instead displaying an intermediate colonization defect (Fig. 5). The *B. pertussis* ∆*cyaC* was similarly not as defective as the ∆*cyaA* mutant in mice (Fig. 6). These data indicate that non-acylated ACT is at least partially functional during infection and are consistent with the fact that although for both *B. bronchiseptica* and *B. pertussis*, the ∆*cyaC* mutant was severely defective relative to the wild-type strain for intoxication of J774 macrophage-like cells, it was still able to intoxicate to a low level (Fig. 3). Use of the K983R and K860R mutants showed that although both are required for hemolysis, only K860 is required for full persistence in mice (Fig. 6), indicating that, in *B. bronchiseptica* at least, acylation of K983 is not required for ACT functionality during infection. These data also support the conclusion that pore formation by ACT may play only a minor role during infection (34).

Previous studies have shown that the inflammation induced by *B. bronchiseptica* is primarily mediated by neutrophils, which are critical for controlling infection at early time points (27). Our data are consistent with a scenario in which non-acylated ACT can inhibit the neutrophilic response in the initial stage of infection (days 1 and 3) but cannot overcome the macrophage-mediated response later in infection, as illustrated by the *B. bronchiseptica* ∆*cyaC* defect being more dramatic when the immune response is more macrophage-mediated (days 11 and 21). In contrast to the case with *B. bronchiseptica*, neutrophils are not critical for controlling *B. pertussis* infection in mice (42). In fact, the early recruitment of neutrophils in *B. pertussis* mouse infection is suppressed by pertussis toxin (PTX) (43), which *B. bronchiseptica* does not produce. Production of PTX by *B. pertussis* could also at least partially account for the fact that we observed less inflammation induced by *B. pertussis* than *B. bronchiseptica* during infection in mice (Fig. 5B and 7B). Our data support the hypothesis that *B. bronchiseptica* produces larger quantities of less acylated ACT to defend against neutrophils, while *B. pertussis* has evolved to secrete smaller quantities of fully acylated ACT to be able to defend against other cell types because it produces PTX to defend against neutrophils.

This study highlights the importance of conducting comparative analyses between closely related species to better understand how evolution has shaped virulence. By studying *B. bronchiseptica* in a natural-host model, we can uncover molecular mechanisms of disease in natural host-pathogen interactions. Through direct comparison with *B. pertussis*, which we do not have natural-host models for, we can appreciate nuanced differences that could impact pathogenicity.

## MATERIALS AND METHODS

### Bacterial growth conditions

*Bordetella bronchiseptica* strains were grown on Bordet-Gengou agar (BD Biosciences) supplemented with 6% or 12.5% defibrinated sheep’s blood (HemoStat Laboratories) as notated and grown at 37°C for 2–3 days. *Bordetella pertussis* strains were grown on Bordet-Gengou agar (BD Biosciences) supplemented with 12.5% defibrinated sheep’s blood (HemoStat Laboratories) and grown at 37°C for 3–4 days. For liquid cultures, *Bordetella* strains were grown in SS medium (44; updated in reference 45) at 37°C overnight. As needed, media was supplemented with streptomycin (20 µg/mL), kanamycin (50 µg/mL), CaCl_2_ (1.8 mM), or MgSO_4_ (50 mM). *Escherichia coli* strains were grown in lysogeny broth (LB) or on LB agar at 37°C. As needed, media was supplemented with kanamycin (50 µg/mL), ampicillin (100 µg/mL), or diaminopimelic acid (300 µg/mL).

### Plasmid and strain construction

A detailed strain and plasmid list can be found in Table S1. *E. coli* strain DH5⍺ was used to construct and amplify plasmids, and *E. coli* strain RHO3 was used for conjugation to *B. bronchiseptica* and *B. pertussis*. In-frame deletions were constructed in *B. bronchiseptica* and *B. pertussis* via allelic exchange using derivatives of the pSS4245 vector, and complementation strains were created in *B. bronchiseptica* and *B. pertussis* via transposase-mediated insertion at the *att*Tn*7* site using derivatives of the pUC18 vector. All mutations were confirmed by PCR and/or sequencing.

### Immunoblots

Samples were prepared from overnight cultures grown in modified SS containing 2 mM Ca^2+^ for 16 hours that reached a final OD_600_ of ~3–4. Whole cell lysate samples were prepared by pelleting a volume equivalent to 1 OD_600_ of culture and boiling in Laemmli buffer. Supernatant samples were taken from the same culture tubes, and 2 mL of culture supernatant was filtered through 0.2 µm filters. Ten percent trichloroacetic acid was used to precipitate proteins, and the pellets were rinsed with acetone before being resuspended in Laemmli buffer mixed with 1 M Tris-HCl, pH 8.8, at a volume normalized to OD_600_s and boiled. Proteins were separated using 4-12% Tris-glycine gradient gels (Invitrogen) and transferred to nitrocellulose membranes (GE Healthcare). Dot blot samples were prepared from overnight cultures grown in modified SS containing 2 mM Ca^2+^ by washing cells with PBS and normalizing samples to an OD_600_ equivalent to 0.5. A total of 100 µL of normalized samples was spotted onto nitrocellulose membranes (GE Healthcare) using a 96-well vacuum manifold.

Membranes were stained with Revert 700 Total Protein Stain (LI-COR Biotech), imaged on a LI-COR Odyssey DLx Imager (LI-COR Biotech), and destained. Membranes were then immunoblotted using mouse monoclonal antibody 9D4 generated against ACT residues 1156–1489 (supplied courtesy of F. Heath Damron) (46). α-Mouse IRDye secondary antibody (LI-COR Biotech) was used for protein detection, and immunoblots were imaged on a LI-COR Odyssey DLx Imager (LI-COR Biotech). Empiria Studio Software v.3.2 (LI-COR Biotech) was used to analyze protein abundances. ACT signals in whole cell lysates and supernatants were normalized to the signal of a protein of equal abundance present in whole cell lysates across all strains detected by Revert 700 Total Protein Stain. Relative abundance values were calculated relative to wild-type *B. bronchiseptica* values and averaged from two biologically independent experiments. Representative images are shown of at least three biologically independent experiments.

### J774A.1 cell intoxication assays

J774A.1 cells (ATCC) were grown in Dulbecco’s modified Eagle medium with high glucose and pyruvate (Thermo Fisher), supplemented with 10% fetal bovine serum (VWR), 2 mM L-glutamine (Gibco), and 1% MEM Non-Essential Amino Acids (Gibco). For the intoxication assay, J774A.1 cells were seeded at 1 × 10^6^ cells per well. Seed media was removed and replaced with growth media containing bacterial cells at an MOI of 100 with an *n* = 2. Plates were centrifuged for 5 minutes at 500 × *g*, then incubated for 30 minutes at 37°C in 5% CO_2_. Cells were rinsed with Dulbecco’s phosphate-buffered saline (DPBS) (Gibco), then lysed with 0.1 M HCl with 0.5% Triton X-100 for 20 minutes. Lysates were centrifuged at 21,000 × *g* for 10 minutes to remove cell debris, and intracellular cAMP-containing supernatants were used in the competitive cAMP ELISA (ENZO). cAMP concentrations (pmol/mL) were determined according to the manufacturer’s protocol (ENZO) and were adjusted by subtracting the uninfected control.

### Mass spectrometry

Samples were prepared by purification of ACT from supernatants of *B. pertussis* and *B. bronchiseptica* strains grown overnight in SS liquid broth cultures as described in the immunoblots section. Proteins were separated by size on a 4–12% Bis-Tris gradient gel (Invitrogen). Bio-Safe Coomassie G-250 Stain (Bio-Rad) was used to stain the gel to visualize proteins, and the Coomassie-stained gel was submitted to the UNC Proteomics Core. Gel bands corresponding to ACT by size were excised and de-stained overnight. Samples were reduced with 10 mM dithiothreitol (DTT), alkylated with 100 mM iodoacetamide, and digested with trypsin overnight. Digested peptides were cleaned using C18 desalting spin columns (Pierce). Samples were analyzed in technical duplicates by LC-MS/MS using a Thermo Easy nLC 1200-QExactive HF, and data analysis was conducted in Perseus. Proteome Discoverer (Thermo Scientific; v.2.5) was used to search the data against the *Bordetella bronchiseptica* proteome from Uniprot and a common contaminants database (245 sequences). Results were filtered for 1% false discovery rate (FDR), and relative abundances of peptides were corrected using a global normalization to account for differences in protein abundances between samples.

### Mouse inoculations

Six-week-old BALB/c mice from Charles River Laboratories (catalog no. BALB/cAnNCrl) were intranasally inoculated with 1 × 10^5^ CFU *B. bronchiseptica* or 1 × 10^6^ CFU *B. pertussis* in 50 µL of DPBS. The trachea and right lung lobe were harvested from mice into 1 mL DPBS at indicated time points post-inoculation. Tissues were homogenized, and CFU were enumerated by plating serial dilutions on Bordet-Gengou agar supplemented with streptomycin.

### Histological analysis

Left lung lobes were harvested from mice at day 3 post-inoculation and inflated with 10% formalin (Sigma-Aldrich). Lung tissues were embedded in paraffin, sectioned 5 µm thick, and stained with H&E by the UNC Histology Research Core Facility. Prepared slides were examined using bright-field imaging on a Keyence BZ-X810.

## SUPPLEMENTAL MATERIAL

Table S1 mbio.01082-25-s0001.pdf Strains and plasmids.

ASM does not own the copyrights to Supplemental Material that may be linked to, or accessed through, an article. The authors have granted ASM a non-exclusive, world-wide license to publish the Supplemental Material files. Please contact the corresponding author directly for reuse.
