# Supplementary material for: Nuanced differences in adenylate cyclase toxin production, acylation, and secretion may contribute to the evolution of virulence in Bordetella species
Source: mBio. 2025 May 19;16(6):e01082-25. doi: 10.1128/mbio.01082-25 (PMC12153271; doi:10.1128/mbio.01082-25)
Supplement: Table S1 — Strains and plasmids. [file mbio.01082-25-s0001.pdf]

Table S1. Bacterial strains and plasmids used in this study

Bacterial strains	Description	Reference
B. bronchiseptica strains		
RB50	Wild-type *B. bronchiseptica* Complex 1 strain	(1)
RB515 (Δ*cyaA*)	RB50 containing a deletion of *cyaA* codons 5-1701 using pCI56	(2)
RB524 (Δ*cyaC*)	RB50 containing a deletion of *cyaC* codons 4-183 using pCI62	This study
Δ*cyaC*,*cyaC*^{C}	RB524 containing *nptII* (Km^{r}) and 477bp upstream *cyaC* through *cyaC* coding sequence at the *att*Tn*7* site using pAW16	This study
RBX24 (Δ*fhaB*Δ*sphB1*)	RBX9 containing a deletion of *sphB1* codons 5-1035	(3)
RBX24 Δ*cyaC*	RBX24 containing a deletion of *cyaC* codons 4-183 using pCI62	This study
RBX24 *cyaC*++	RBX24Δ*cyaC* containing *nptII* (Km^{r}) and *cyaC* driven by the S12 promoter at the *att*Tn*7* site using pAW12	This study
RB509 (iACT)	RB50 containing the H63A, K65A point mutations in ACT resulting in catalytically inactive ACT	(2)
ACT-K860R	RB50 containing the K860R point mutation in ACT constructed by allelic exchange using pAW14	This study
ACT-K983R	RB50 containing the K983R point mutation in ACT constructed by allelic exchange using pAW26	This study
ACT-K860R+K983R	RB50 containing the K860R, K983R point mutations in ACT constructed by allelic exchange using pAW33	This study
B. pertussis strains		
BPSM	Wild-type *B. pertussis* Tohama I derivative	(4)
Bp536	Wild-type *B. pertussis* Tohama I derivative	(5)
Bp5362 (Δ*cyaA*)	Bp536 containing a deletion of *cyaA* codons 5-1701 constructed by allelic exchange using pCI46a	This study
Δ*cyaC*	BPSM containing a deletion of *cyaC* codons 4-183 constructed by allelic exchange using pAW23	This study
Δ*cyaC*,*cyaC*^{C}	BPSM Δ*cyaC* containing *nptII* (Km^{r}) and 477bp upstream *cyaC* through *cyaC* coding sequence at the *att*Tn*7* site using pAW32	This study
Δ*sphB1*	BPSM containing a deletion of *sphB1* codons 5-1035	(6)
Bpe138	Bp536 containing a gene conferring Cm^{r} in place of *fhaB*	(7)
Bp1381 (Δ*fhaB*Δ*sphB1*)	Bpe138 containing a gene conferring Cm^{r} in place of *fhaB* and a deletion in *sphB1* codons 5-1035 using pSS1129Δ*sphB1*	This study
E. coli strains		
DH5α	Molecular cloning strain	ThermoFisher
RHO3	Conjugation strain (DAP auxotroph)	(8)
Plasmids		
pSS4245	Allelic exchange plasmid used for construction of in-frame deletions in *Bordetella* species; Ap^{r} Km^{r} Smr	(9)

pSS1129	Allelic exchange plasmid containing *rpsL* used for construction of in-frame deletions in *B. pertussis*	(10)
pUC18-mini-Tn*7*-km	Mobilizable transposition vector; Ap^r Km^r	(11)
pTNS3	Tn*7* transposase expression vector containing *tnsABCD*; Ap^r	(11)
pUCS12	pUC18-mini-Tn*7*-km used to integrate genes of interest driven by the S12 promoter at the *att*Tn*7* site; Ap^r Km^r	(11)
pSS1129Δ*sphB1*	pSS1129 derivative used to delete codons 5-1035 in *sphB1*$_{Bp}$	(6)
pCI46a	pSS1129 derivative used to delete codons 5-1701 in *cyaA*$_{Bp}$	This study
pCI62	pSS4245 derivative used to delete codons 4-183 in *cyaC*$_{Bb}$	This study
pAW12	pUCS12 derivative used to integrate *cyaC*$_{Bb}$ driven by the S12 promoter at the *att*Tn*7* site	This study
pAW14	pSS4245 derivative used to change *cyaA*$_{Bb}$ to encode K860R	This study
pAW16	pUC18-mini-Tn*7*-km derivative used to integrate *cyaC*$_{Bb}$ driven by the native *cyaC* promoter (477bp upstream intergenic region) at the *att*Tn*7* site	This study
pAW23	pSS4245 derivative used to delete codons 4-183 in *cyaC*$_{Bp}$	This study
pAW26	pSS4245 derivative used to change *cyaA*$_{Bb}$ to encode K983R	This study
pAW32	pUC18-mini-Tn*7*-km derivative used to integrate *cyaC*$_{Bp}$ driven by the native *cyaC* promoter (477bp upstream intergenic region) at the *att*Tn*7* site	This study
pAW33	pSS4245 derivative used to change *cyaA*$_{Bb}$ to encode K860R+K983R	This study

1. Cotter PA, Miller JF. 1994. BvgAS-mediated signal transduction: analysis of phase-locked regulatory mutants of *Bordetella bronchiseptica* in a rabbit model. Infect Immun 62:3381–3390.

2. Henderson MW, Inatsuka CS, Sheets AJ, Williams CL, Benaron DJ, Donato GM, Gray MC, Hewlett EL, Cotter PA. 2012. Contribution of *Bordetella* Filamentous Hemagglutinin and Adenylate Cyclase Toxin to Suppression and Evasion of Interleukin-17-Mediated Inflammation. Infect Immun 80:2061–2075.

3. Nash ZM, Inatsuka CS, Cotter PA, Johnson RM. 2024. *Bordetella* filamentous hemagglutinin and adenylate cyclase toxin interactions on the bacterial surface are consistent with FhaB-mediated delivery of ACT to phagocytic cells. mBio 15:e00632-24.

4. Menozzi FD, Mutombo R, Renauld G, Gantiez C, Hannah JH, Leininger E, Brennan MJ, Locht C. 1994. Heparin-inhibitable lectin activity of the filamentous hemagglutinin adhesin of *Bordetella pertussis*. Infect Immun 62:769–778.

5. Relman DA, Domenighini M, Tuomanen E, Rappuoli R, Falkow S. 1989. Filamentous hemagglutinin of *Bordetella pertussis*: nucleotide sequence and crucial role in adherence. Proc Natl Acad Sci 86:2637–2641.

6. Mazar J, Cotter PA. 2006. Topology and maturation of filamentous haemagglutinin suggest a new model for two-partner secretion. Mol Microbiol 62:641–654.

7. Inatsuka CS, Julio SM, Cotter PA. 2005. *Bordetella* filamentous hemagglutinin plays a critical role in immunomodulation, suggesting a mechanism for host specificity. Proc Natl Acad Sci 102:18578–18583.

8. López CM, Rholl DA, Trunck LA, Schweizer HP. 2009. Versatile Dual-Technology System for Markerless Allele Replacement in *Burkholderia pseudomallei*. Appl Environ Microbiol 75:6496–6503.

9. Inatsuka CS, Xu Q, Vujkovic-Cvijin I, Wong S, Stibitz S, Miller JF, Cotter PA. 2010. Pertactin Is Required for *Bordetella* Species To Resist Neutrophil-Mediated Clearance. Infect Immun 78:2901–2909.

10. Stibitz S, Black W, Falkow S. 1986. The construction of a cloning vector designed for gene replacement in *Bordetella pertussis*. Gene 50:133–140.

11. Anderson MS, Garcia EC, Cotter PA. 2012. The *Burkholderia bcpAIOB* Genes Define Unique Classes of Two-Partner Secretion and Contact Dependent Growth Inhibition Systems. PLoS Genet 8:e1002877.
